# Why Current Detection of Vascular Calcification Falls Short and How to Improve on It

**DOI:** 10.1055/a-2495-1444

**Published:** 2024-12-27

**Authors:** Anouk Gentier, Mueez Aizaz, Maurice Halder, Alexandru Florea, Ingrid Dijkgraaf, Felix M. Mottaghy, Tilman Hackeng, M Eline Kooi

**Affiliations:** 1 Department of Biochemistry, Cardiovascular Research Institute Maastricht (CARIM), University Maastricht, Maastricht, The Netherlands; 2 Cardiovascular Research Institute Maastricht (CARIM), Maastricht University, Maastricht, The Netherlands; 3 Department of Radiology and Nuclear Medicine, Maastricht University Medical Center, Maastricht, The Netherlands; 4 Department for Renal and Hypertensive, Rheumatological and Immunological Diseases (Department of Medicine II), RWTH Aachen, Medical Faculty, Aachen, Germany; 5 Department of Nuclear Medicine, University Hospital RWTH Aachen, Aachen, Germany

**Keywords:** imaging, cardiovascular disease, noninvasive imaging, early detection, vascular calcification

## Abstract

Vascular calcification is a common phenomenon in various vascular diseases, where its presence heralds increased occurrence of adverse disease events, which invariably lead to increased morbidity and mortality in patients. Although the impact of calcification has become apparent, adequate and early detection of the most damaging form of early microcalcification is still in its infancy, preventing reliable identification of locations that would benefit from intervention. In this review, we will provide an overview of the current state-of-the-art noninvasive calcification imaging and its persisting limitations. We discuss promising approaches that may address these limitations in the future. In this context particular attention will be paid to imaging modalities such as CT, PET, and ultrasonography and molecular and cellular mechanisms and agents involved in physiological bone formation.

## Introduction

Each year, over 18 million people worldwide suffer from acute myocardial infarction, stroke, or consequences of peripheral thrombi due to complications caused by atherosclerosis and arterial stiffness and their associated forms of vascular calcification, intimal and medial calcification respectively. 1 2 3

The process of atherosclerosis is characterized by the interplay of lipid metabolism, active cellular interactions, inflammation, and extracellular matrix (ECM) remodeling. 4 5 The development of atherosclerosis is initiated by persistent endothelial activation. 5 6 This endothelial activation can be induced by various stimuli, ranging from the results of the natural aging process and hereditary diseases to chronic inflammatory illnesses and their accompanying structural and functional changes. 3 6 These endothelial changes lead to exposure of the underlying collagen layer to the blood, resulting in platelet adherence and aggregation. 6 The adhering platelets in turn release chemokines, interleukins, and several other inflammatory factors that serve as homing beacons and activators for macrophages and lymphocytes. 7 8 9 The cytokines, produced by the recruited immune cells, along with platelet and plasma factors will help maintain the inflammatory microenvironment that drives atherosclerotic plaque development. 7 10 Although inflammation is a key feature of developing atherosclerotic plaques, at the same time, repair processes will try to resolve the sustained vessel wall injury. 10 11 12 These processes, however, are not potent enough to truly repair the damage and will attempt to isolate the inflamed and necrotic thrombogenic arterial wall from the circulating blood by formation of a fibrous cap. 10

Every healthy individual will experience some degree of atherogenesis during aging and, consequently, carry atherosclerotic lesions within their vasculature. 13 14 Most of the time atherosclerotic lesions will remain subclinical up until the plaque becomes vulnerable to rupture or erosion and subsequent thrombus formation. 15 In the prevention of adverse vascular events, early detection of this loss of stability is key.

Rupture of an atherosclerotic plaque can best be described as a mechanical event caused by the local pressure on the vessel wall exceeding the local tensile strength of the affected tissue, where both the local pressure on the tissue as well as its strength are dependent on plaque composition. 16 Plaque composition is determined by the type of atherogenic process that is dominant at that point in time. In clinical practice, fibrous cap thickness, presence of intraplaque hemorrhage, necrotic core size, persistent inflammation, and calcification state are the go-to plaque components used to determine plaque vulnerability. 17 18 19 20 Erosion of an atherosclerotic plaque, in turn, is best defined as the pro-thrombotic result of intimal layer denudation caused by increased wall shear stress through disturbed flow, endothelial dysfunction, neutrophil recruitment, and subsequent release of neutrophil extracellular traps (NETosis). 21 22

Clinically, coronary artery calcium score, as determined by computed tomography (CT), is considered the most reliable and independent indicator of overall atherosclerotic burden and risk determinant of adverse coronary events. 23 24 Stroke risk, however, is strongly determined by the presence of intraplaque hemorrhage in carotid plaques as scored on magnetic resonance imaging (MRI). 25 26 However, in recent years detection of microcalcifications, defined as calcifications with a diameter below 50 µm, with positron emission tomography (PET) using radioactive sodium [ ^18^ F]fluoride (Na[ ^18^ F]F), has also emerged in the field of cardiovascular imaging to visualize active calcification, which has been linked to increased risk of adverse cardiovascular events. 27 28 29 30 31 32

Given that these imaging techniques have inherent limitations, and particularly for Na[ ^18^ F]F PET doubts remain regarding its effectiveness in identifying rupture-prone plaques, better noninvasive detection methods that are more specific for vascular calcification are needed. In this position paper our aim is twofold: to provide an overview of recent developments in the field of noninvasive medical imaging in the context of vascular calcification in atherosclerosis and to identify new biologically relevant sources for the design and development of calcification-specific molecular imaging tracers.

## Alternative Imaging Modalities

**Fig. 1:**
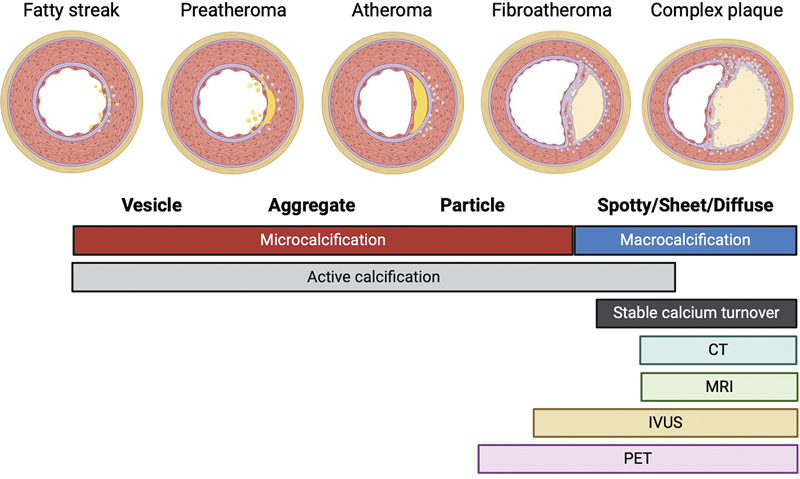
Overview of developmental stages of atherosclerotic calcification, incorporating developmental stages, classification of calcification type, and detection scope of currently available imaging modalities. CT, computed tomography; IVUS, intravascular ultrasound; MRI, magnetic resonance imaging; PET, positron emission tomography. (Created with BioRender.com.)

In recent years, the quality, reliability, and variety of available noninvasive imaging techniques for identification of the vulnerable atherosclerotic plaque have grown through advances in both hardware and software. These advances have provided many solutions to inherent limitations of CT and Na[ ^18^ F]F PET, particularly with regard to detection of vascular calcification ( Fig. 1 ).

### Computed Tomography

CT enables the reconstruction of images based on the X-ray attenuation characteristics of different tissues. Calcified tissues exhibit high attenuation and thus appear bright on CT images. For CT, the main shortcomings are insufficient spatial resolution for detection of early, high-risk calcification events, vulnerability to blooming artefacts, distortions caused by small, high-density structures within the tissue (e.g., vascular calcification, metal stent), and overlap in attenuation between the calcification and the iodine contrast agent. 31 33 34 35 The consequence of this vulnerability of CT to blooming artefacts is a tendency to overestimate the calcified volume in the vasculature of the heart or the carotid arteries during cardiac or carotid CT angiography (CCTA). 31 33 34 The overlap in attenuation, which is expressed in Hounsfield units, between the calcification and the iodine contrast agents also interferes with accurate detection of vascular calcification. 31 33 35 In human studies, radiation exposure and the necessity of iodine-based contrast agents in diagnostic images of the coronary arteries is a further drawback to take into consideration. 31 36

However, several of these inherent drawbacks of CT may be overcome by photon-counting CT. 37 This novel clinical CT system allows for precise detection of incident energy generated by individual X-ray photons, which leads to a reduced radiation exposure, and an improved spatial resolution as well as an improved contrast-to-noise ratio (CNR) and the ability to better distinguish iodine-based contrast from calcifications in comparison to conventional CT. 37

Although the spatial resolution of clinical CT, including photon-counting CT, is sufficient to detect macrocalcification (>50 µm) that is present in later stages of atherogenesis as well as some other high-risk plaque features, it cannot detect the early-stage microcalcification (<50 µm) that gives rise to plaque instability. 31 32

In preclinical CT systems, used for *in vivo* and *ex vivo* studies, spatial resolutions of 10 µm to less than 1 µm have been achieved, respectively. 38 39 Any form of calcification has an adverse effect on the stress resistance of the surrounding tissue, with microcalcification proving to be to be a reliable indicator of an increased rupture risk. 16 40 Microcalcification holds potential for successful intervention toward increasing plaque stability and improving disease outcome, as this early-stage plaque feature can be approached from several angles. Anti-calcification interventions can directly target the forming nidi or existing calcification deposits, by interfering with intraplaque inflammation or by modulating vascular smooth muscle cell (VSMC) phenotypic switching toward a plaque resolving profile. 3 Each of these approaches necessitate specific detection of this form of vascular calcification, currently best performed by Na[ ^18^ F]F PET. 41 42

### Na[ ^18^ F]F PET

Initially, Na[ ^18^ F]F PET was utilized for bone scanning in the context of bone turnover and detection of bone tumors. 43 Recently, Na[ ^18^ F]F PET has found further application in vascular calcification risk assessment. 27 44

The challenges with Na[ ^18^ F]F PET–based calcification burden determination are two-sided, stemming from both the inherent limitations of PET and those associated with the tracer Na[ ^18^ F]F. PET itself is a noninvasive imaging modality where specific radioactive tracers are used to visualize various biochemical features. 45 Depending on the radiotracer used, PET allows for a high target-to-background ratio, resulting in a high CNR to the surrounding tissues, in turn necessitating the use of other imaging modalities (e.g., CT, MRI) to enable identification of untargeted anatomical features. 46 47 The use of specific tracers allows for targeted and highly sensitive assessment of the process in question. 45 However, this need for tracers also poses a drawback in that detection of a specific target process can only occur if an appropriate tracer exists. For Na[ ^18^ F]F, calcification is detected by the exchange of the radioactive fluoride ( ^18^ F ^-^ ) with the hydroxyl group present in hydroxyapatite, thereby labelling all hydroxyapatite crystals. Moreover, in the context of vascular calcifications, Na[ ^18^ F]F is able to more effectively target active sites of microcalcifications, which are not detected by CT, due to the inability of fluoride to penetrate thick crystals and therefore having a more pronounced uptake in regions with small and diffuse developing calcifications. 48 Notably, Na[ ^18^ F]F is not able to differentiate between physiological and pathological forms of calcification as it only detects the end product of a calcification process irrespective of its underlaying nature. 17 49 This inability creates challenges when the vascular beds of interest are near bone, making it unsuitable for direct study of underlying processes that specifically drive vascular calcification, which are currently not fully understood.

### Ultrasound

An alternative to assessing vascular calcification with conventional techniques of CT or PET is found in ultrasound (US). 50 In particular, the more advanced techniques such as contrast-enhanced ultrasound (CEUS) and intravascular ultrasound (IVUS) provide viable alternatives for the detection of hallmarks of vulnerable atherosclerotic plaques. 50 51 Of these modalities, IVUS is currently hailed as providing near-histology quality images of the vascular wall and plaque make-up, including sufficient sensitivity and specificity for detection of vascular calcification. However, IVUS is not without limitations, as the technique itself requires an invasive catheterization procedure, detects only dense calcium deposits (i.e., macrocalcifications), and is unable to calculate calcification thickness due to its inability to penetrate calcium deposits, thus hampering risk prediction by IVUS. One approach to improve IVUS risk prediction is the development of automated calcium detection algorithms. 52

A possible approach to enable detection of lower density calcifications with US is CEUS. This specialized US technology relies on microbubble-based contrast agents to increase vessel visibility by improving the CNR of the acquired images. 51 53 54 55 The microbubbles enable this improvement by their nonlinear response to US, which increases amplitudes of transmitted US waves. 51 This nonlinear response of the microbubbles entails that at exposure of the bubbles to the component of the US wave that exudes rarefactional pressure, the bubbles expand in response, but when they encounter the compression component of this same US wave the bubbles only barely contract. 51 Due to these different responses to changes in pressure, the microbubbles generate a harmonic frequency with an amplitude significantly higher than those produced by the surrounding tissues, thus making the microbubbles clearly distinguishable. 51

Currently, the enhancement of image quality by CEUS has proven its worth in detecting plaque-associated neovascularization and plaque inflammation. 54 56 In time it is expected that this US technique can be extended to allow detection and possible treatment of microcalcification structures. One approach to achieve this could be to decorate the surface of the bubble with proteins, peptides, or antibodies, which home in on suspected sites of active calcification. These decorations could then either guide and accumulate the microbubbles at areas of vascular calcification or be released from the surface and enter into the vessel wall, at predetermined locations of interest. 57 Alternatively, compounds could also be loaded into the microbubbles for transport to suspected high-risk plaques, where their contents will be released. 58 59 The abovementioned release of tracer or treatment is accomplished through disintegration of the bubbles by US through a process called sonoporation. 58 59 Sonoporation has as additional advantage that it increases the permeability of the local tissues for the released compounds. 60 The most significant disadvantage of this US method is its limited penetration depth into tissue, making it primarily suited for assessment of superficial blood vessels and resulting in the need for an invasive approach when used for deeper parts of the vasculature. Other limitations associated with CEUS include operator dependence, as image quality is greatly influenced by operator skill, and its difficulty in visualizing tissues behind areas of calcification similar to IVUS. Furthermore, it is currently still unable to distinguish intraplaque hemorrhage from lipid deposition. Finally, there is also the risk of plaque rupture when sonoporation is used in conjunction with CEUS to deliver local treatment.

### Hybrid Imaging Systems

A promising approach for the detection of vascular calcification is the use of hybrid imaging systems, which combines two conventional imaging modalities so that the shortcomings of one technique are compensated by the other. Examples of such hybrid systems are PET/CT and PET/MRI. 61 62

In both the techniques the PET component provides a solution to the limited sensitivity of CT and MRI with its highly sensitive, radioactively labelled tracers. In turn, the CT or MRI component of the multimodal set-up provides a detailed anatomical image of the assessed region. It should be noted that although contrast agents detectable by X-ray or MRI exist, which allow for more opaque features of the atherosclerotic plaque to be highlighted, these are neither approved for use in humans nor specifically geared toward microcalcification. 57

As far as the PET component of these hybrid systems is concerned, Na[ ^18^ F]F has a firm monopoly as the go-to tracer in research settings for detection of small (<50 µm), ongoing calcification events. 32 A significant limitation of Na[ ^18^ F]F in vascular calcification assessment, beyond its detection of both physiological and pathological calcification, is its equal take up by both unstable, unruptured and recently ruptured lesions. 17 63 This uptake by ruptured plaques creates difficulties in pinpointing which areas would benefit most from rupture prevention as well as in performing accurate risk assessment for cardiovascular events. For this reason, development and application of novel imaging modalities for detection of vascular calcification as well as new, specific, process-associated microcalcification tracers are crucial.

## Biological Bone Tracers

The development of new microcalcification tracers has been greatly aided in recent years by progress in the field of nanotechnology. 57 Currently, several approaches, including Na[ ^18^ F]F PET, are under development for the direct detection of CT-invisible microcalcifications. 42 44 48 One of these new approaches is the use of the bisphosphonates, originally a class of drugs used to treat osteoporosis, as the basis for new tracers. An example of such a tracer is [ ^64^ Cu]Cu-DOTA-alendronate. 64 This tracer, although initially developed for use in preliminary risk assessment in breast cancer, has shown to be capable of sensitive and specific detection of hydroxyapatite-based microcalcifications in an age-related breast cancer rat animal model. 64 As such, it can be argued that [ ^64^ Cu]Cu-DOTA-aldronate could have potential as a tool for the detection of vascular microcalcifications comparable to Na[ ^18^ F]F. Another bisphosphonate-derived agent that is being used for both *in vitro* and *ex vivo* detection of early-stage calcification is fluorescein-bisphosphonate conjugate 1. 65 This bisphosphonate-based tracer showed a greater sensitivity and specificity for hydroxyapatite calcification when compared to gold standards like Alizarin S, Na[ ^18^ F]F, and CT. 65

Nanomaterials are another approach related to the use of previously mentioned nanotechnology for detection of calcification centers. More specifically, the use of liposomes and micelles decorated with peptides, antibodies, or nanobodies against calcification-associated biomarkers. 57 An example of this is the micellar-based HAP-PAM-Cy7 tracer. 66 This tracer is composed of a peptide amphiphile micelle (PAM), decorated with hydroxyapatite binding peptide (HAP) and labelled with Cy7, for detection *via* fluorescence. 66 Assessment of this novel calcification tracer *in vitro* , on mouse aortic smooth muscle cells, *ex vivo* , on calcified human arteries, and *in vivo* , through injection in live, western diet fed ApoE ^−/−^ mice followed by ex vivo imaging of their aortas, showed specific detection of calcified regions. 66

All previously described tracers, however, still rely on the detection of formed or forming hydroxyapatite deposits instead of the underlying biology that governs the calcification process, making it more difficult to prevent—or intervene with—calcification, as detection requires some level of established calcification. 67 This underlines the necessity to further unravel the molecular mechanisms of vascular calcification to meet the need for new vascular calcification specific tracers that target components preceding the calcification process. Furthermore, the current reliance on the presence of hydroxyapatite within the plaque also entails that the patient is already at significant risk of experiencing severe adverse cardiovascular events. 23 In the following sections of this paper, we discuss new avenues for detection and tracer development as well as highlight new targets ( Table 1 ).

**Table 1:** Summary of all potential targets for calcification-specific tracer development based on involvement in the calcification process

Family	Name	Location	Function	Application	References
Annexins					
	ANXA2	Calcifying VSMC EVs	Enhanced uptake of Ca, colocalizes with TNAP, part of calcification nucleation complexes w/o reliance on channel function	Target	78 80 84
	ANXA5	Calcifying VSMC EVs	Formation of calcification nucleation complex PS-S100A9-ANXA5	Target	78 80
	ANXA6	Calcifying VSMC and its derived EVs	Enhanced uptake of Ca, colocalizes with TNAP, part of calcification nucleation complexes w/o reliance on channel function	Target	80 84
Bone Morphogenic proteins					
	BMP2	VSMC, pericytes, myofibroblast, monocytes	Osteogenic & chondrogenic differentiation	Tracer	68 69
	BMP4	VSMC	Osteogenic & chondrogenic differentiation	Tracer	68 69
	BMP6	Endothelium, VSMC	Osteogenic & chondrogenic differentiation	Tracer	68 69
	BMP7	VSMC	Protective against calcification: inhibits proliferation and stimulates expression of contractile VSMC markers (in vitro)	Treatment	68 69 98 99
Calgranulins/ S100 proteins					
	S100A8	Macrophages, foam cells, neutrophils	Indicator of pro-atherogenic phenotype	Target	85 87 88
	S100A9	Monocytes, macrophages, foam cells, neutrophils, extracellular matrix, matrix vesicles	Indicator of pro-atherogenic phenotype, found expressed on MF and FC located close to calcified areas	Target	85 87 88
	S100A12	Endothelium, VSMC, macrophages, neutrophils	Involved in inflammatory signaling/pro-atherogenic cascade, in situ expression during atherosclerosis has greater impact on vascular calcification than when in circulation	Target	85 88 89
Fetuins					
	Fetuin A	VSMC-derived EVs	Plasma carrier protein for calcium and phosphate; negative regulator of bone & calcium metabolism (occurs *via* formation of calciprotein particles)	Tracer Treatment	84 94 95 96 97
Gla proteins					
	MGP	VSMC and VSMC-derived EVs	Protective against calcification; uncarboxylated MGP colocalizes to places of vascular calcification; antagonist of BMP2	Target Tracer Treatment	100 102 103 105
	Osteocalcin	Osteoblast-like VSMC	Stimulator of osteogenic differentiation and mineralization; assists in incorporation of calcium into ECM	Tracer Treatment	109
	Coagulation factors	VSMC-derived EVs	Select coagulation factors have a protective effect against calcification and show the ability to home to sites of calcification	Target Tracer Treatment	100 101
Phosphatase					
	TNAP	Osteoblast-like VSMC and their derived EVs	Propagation of hydroxyapatite crystals onto the collagen extracellular matrix	Target	91

Abbreviations: ECM, extracellular matrix; EVs, extracellular vesicles; FC, foam cells; MF, macrophages; MGP, matrix Gla protein; TNAP, tissue-nonspecific alkaline phosphatase; VSMC, vascular smooth muscle cell.

## Novel Avenues for Development of Vascular Calcification Tracers

A valuable approach for the development of new tracers for vascular calcification may lie in analyzing molecular mechanisms of normal bone formation, a process which is foreign to the vasculature under normal conditions. As the mechanisms of vascular calcification are better understood, it has become clear that this pathological process shares several characteristics with regular bone formation. 68 Consequently, findings from bone formation could yield novel markers for early vascular microcalcification. A first shared feature of these processes is the involvement of specialized cell types to guide the calcification processes. 69 For our purpose of detecting early calcification events, osteoblasts and osteoblast-like VSMCs that share certain gene expression patterns are of interest. 70 71 In their respective settings of bone formation and vascular calcification, these cells fulfill the role of calcification matrix producing cells, which is an important prerequisite for occurrence of calcification. 71 The second feature that bone formation and vascular calcification have in common is extracellular vesicles (EVs). 72 73 These EVs serve as a nucleation site for calcification due to their specific membrane and intracellular protein composition or as a means for cell–cell communication. 72 73 Although little is known about similarities between bone EVs and atherosclerotic plaque EVs, there is a high probability that both share the same or highly similar features, either in their intracellular content or in membrane-associated proteins, thereby allowing them to act as calcification nuclei. 73 A final feature in both forms of calcification possesses is the presence of a specific calcification enabling microenvironment that can be influenced by the status of the local immune system. 74

Each of these three shared features between physiological bone formation and pathological vascular calcification has the potential to be utilized in the early detection and treatment of atherosclerotic calcification. However, in the context of this review, we will primarily focus on possible avenues of detecting calcification-contributing VSMC phenotypes and calcification-associated EVs through their similarity with components of physiological bone formation.

As mentioned, both bone ossification and vascular calcification utilize EVs as a means of intercellular communication and focal point for calcium crystal formation. 72 73 Although the function of the ossifying and calcifying EVs can be considered highly similar, the proteomes of these EVs may differ substantially due to the differences in parental cell type. 75 In bone, the EVs are primarily produced by osteoblasts, whereas in vascular disease the pro-calcifying EVs can originate from either leukocytes, erythrocytes, or different subpopulations of VSMCs present at the affected area. 12 76 This difference in parental cell types is reflected in both their membrane and intra-vesicular content composition as well as their effect on disease progression. 76 Since our interest lies in the identification of new targets for early detection of microcalcifications, we will focus on the membrane components of pro-calcifying EVs and phenotypically switched VSMCs present within the atherosclerotic lesion. Notably, because EV membranes reflect features of their parental cell's membrane, only limited discrimination will be possible between potential identifiers of calcification competence of EVs and VSMCs. 75 This lack of distinction is not of great importance because both the EVs and their cells are involved in the initiation and progression of atherosclerotic calcification.

A first source for both targets and tracers for early detection of microcalcification potential within an atherosclerotic lesion is the Annexin family. 77 78 This family of Ca ^2+^ -dependent, phospholipid-binding proteins is involved in various intra- and extracellular biological processes, ranging from mediating membrane structure, exo- and endocytosis, generation of lipid rafts, formation and regulation of ion channels, and cytokinesis to regulation of coagulation, inflammation, apoptosis, and fibrinolysis. 78 Within this family of Annexin, A2 (ANXA2), A5 (ANXA5), and A6 (ANXA6) are the most promising candidates for tracer development, as these ANXAs are expressed within cells derived from either the chondrogenic or osteoblastic lineage as well as being most abundantly present on osteogenic matrix vesicles and actively involved in calcification of these vesicles. 77 78 79 Annexin A5's function in vascular calcification is to form nucleation sites together with S100A9 and phosphatidylserine (S1009A-PS-ANXA5 complex) for initial hydroxyapatite formation, both intra- and extravesicular. 77 80 81 Notably, given ANXA5's established use as a tracer for apoptosis it may be beneficial to focus future tracer development toward the S100A9-PS-ANXA5 complex as opposed to either using ANXA5 as tracer or targeting ANXA5 itself. 82 83 The function of ANXA2 and ANXA6 is to facilitate EV-based microcalcification, both by mediating formation of the vesicles from VSMC and by enabling influx of Ca ^2+^ into the EVs. 80 84

The S100 protein family, as already implied above by the S100A9-PS-ANXA5 complex, also holds promise as a pool for targets and tracers for vascular calcification due to their involvement in various cellular processes, such as proliferation, differentiation, inflammation, migration and invasion, apoptosis, Ca ^2+^ homeostasis, and energy metabolism. 85 86 Particularly the subfamily of calgranulins, S100A8, S100A9, and S100A12, have been observed to play a role in Ca ^2+^ homeostasis and promoting calcification in cardiovascular disease. 85 86 High expression of the calgranulins S100A8 and S100A9 has been well established in numerous inflammatory conditions including atherosclerosis. Recent evidence has also demonstrated that extracellular presence of these calgranulins is associated with the emergence of vascular calcification. 87 It was described that S100A8 served as an inducer for a pro-atherogenic macrophage phenotype, a phenotype that supports the formation of foam cells at sites of atherosclerosis. 87 88 Secondly, it was discovered that S100A9 was abundantly present on macrophages and foam cells located close to sites of calcified deposits, often in complex with ANXA5 and PS. 81 87 Large amounts of S100A9 were also found to be present in matrix vesicles isolated from atherosclerotic carotid arteries and aorta specimens. 87 Taken together, these findings of S100A9 clearly indicate its involvement in intraplaque calcification. Further research is necessary to elucidate the exact role in vascular calcification. In contrast to S100A8 and S100A9, S100A12 shows a much more direct involvement in ectopic calcification as its expression by VSMCs, when exposed to a pro-atherosclerotic environment, leads to an increase of expression of osteogenic phenotype associated genes linked to an observed increase in vascular calcification. 89

Another potential target involved in creating a suitable environment for ectopic calcification to take place is tissue-nonspecific alkaline phosphatase (TNAP). This enzyme is associated with endochondral ossification under physiological conditions and is involved in propagating hydroxyapatite formation, by converting pyrophosphate (PPi) into free phosphate (Pi) creating a more pro-calcifying environment. 90 91 TNAP is an interesting target for detection in the context of assessing the microenvironment surrounding a diseased area as in other fields, like cancer research, it has proven valuable in making an accurate prognosis for disease progression. 92 In the same vein as TNAP, plasma protein fetuin A should also be considered as a valuable target or tracer for early vascular calcification, given its involvement in inflammation, metabolic disease, and mineralization. 93 In the context of biomineralization, fetuin A functions as a mineral chaperone that inhibits unregulated precipitation of calcium–phosphate mineral complexes in plasma by encapsulating them for transport, with the help of other, acidic plasma proteins. 93 94 Due to its function as chaperone of mineralization, fetuin A is abundantly present in areas of both physiological and pathological calcification, a feature that can be exploited to develop a new tracer for early detection and monitoring of vascular calcification. 93 95 96 97

Another valuable resource for the detection of vascular calcification, based on study of physiological bone formation, is the bone morphogenic protein family (BMP). 69 Initially described as osteo-inductive proteins, the BMP family was soon revealed to be critical for normal development and function of various other tissues besides bone. 68 In vascular calcification, BMP2, 4, and 6 are of interest. These cytokines are also members of TGF-β superfamily and strongly associated with plaque vulnerability, osteogenic differentiation, and intraplaque calcification. 69 Another TGF-β family member that could serve as a valuable target is BMP7. 69 This bone morphogenetic protein possesses anti-inflammatory and calcification-protective effects in CKD. 98 99

A final group of proteins worth mentioning as a source for vascular calcification specific tracers and potential theranostics are the vitamin K–dependent post-translationally modified γ-carboxyglutamic acid (Gla)-domain containing proteins (Gla proteins). 100

The value of these Gla proteins as starting point for diagnostic imaging tools as well as potential therapies to prevent or halt progression of calcification within the vasculature lies in their ability to accumulate at sites of calcification and inhibit vascular calcification. 100 101 102 103 Beyond their direct use as a diagnostic or therapeutic agent, Gla proteins could also be used as companion diagnostic during evaluation of other anti-vascular calcification treatments. 96 104 105 106

Within this group, the Gla-domain containing coagulation-associated proteins, like prothrombin and protein S, as well as vascular smooth muscle associated matrix Gla protein (MGP) and osteoblast/osteoblast-like cell-associated osteocalcin, have shown the most promise for development of new, noninvasive calcification tracers and treatment agents due to their inhibiting effect on VSMC calcification and ability to localize to areas of early calcification. 101 106 107

## Conclusion

Of all the processes associated with atherosclerotic plaque vulnerability, none has fewer noninvasive detection options as early-stage intraplaque calcification. In this work, we provided an overview of the recent developments in the field of noninvasive imaging of vascular calcifications both in the clinical and preclinical setting. In addition, we introduced a series of new, more specific calcification targets based on the latest insights on the process of vascular calcification. Taken together, we believe that a large number of new vascular calcification–specific tracers will come into development and clinical use in the coming years. We also expect that many of these new tracers will be designed for use in PET-based imaging due to its high sensitivity and specificity for a designated target, which has already been shown for other pathological or pathology-associated biological processes. 108 This progress will not only allow for earlier detection of at-risk areas that would benefit from either closer monitoring or intervention and new insights into the underlying biological processes driving vascular calcification, but also enable monitoring of therapeutic effectiveness and some may even serve as a combined diagnostic and treatment, a theranostic, to halt or reverse the calcifications themselves. All these facets will, in turn, contribute to an improvement in the quality of life of patients, by preventing adverse vascular events.
